# Allergic sensitization and respiratory infection in infancy—Too early for two‐hits on lung function?

**DOI:** 10.1111/pai.70427

**Published:** 2026-07-21

**Authors:** Vikas Wadhwa, Shyamali C. Dharmage, Caroline Lodge, Jennifer J. Koplin, Katrina J. Allen, Mimi L. K. Tang, Adrian J. Lowe, Rachel L. Peters, Melissa Russell

**Affiliations:** ^1^ Allergy and Lung Health Unit, Centre for Epidemiology and Biostatistics, School of Population and Global Health The University of Melbourne Melbourne Victoria Australia; ^2^ Murdoch Children's Research Institute Melbourne Victoria Australia; ^3^ Child Health Research Centre University of Queensland Brisbane Queensland Australia; ^4^ Department of Paediatrics The University of Melbourne Melbourne Victoria Australia

**Keywords:** allergic sensitization, infancy, interaction, lung function, respiratory infection

## Abstract

**Background:**

Allergic sensitization and respiratory infections are common in childhood. The “two‐hit” hypothesis suggests these interact in early‐life to increase risk of subsequent asthma. The “two‐hit” effect on lung function, however, remains unknown.

**Objective:**

We assessed for interactions between food allergic sensitization and respiratory infection in infancy and associations with lung function in childhood.

**Methods:**

In a longitudinal community‐based cohort, sensitization was assessed at age 1 year by skin prick testing to common food allergens. Respiratory infection was assessed via parental reports of doctor diagnosed bronchiolitis or antibiotics for lower respiratory infection in the first year of life. Regression models were fitted with interactions between these exposures to assess for associations with lung function at ages 6 and 10 years, controlling for potential confounders.

**Results:**

No interaction was observed between food sensitization and lower respiratory infection in infancy on lung function at either age 6 or 10 years. Respiratory infection was associated with modest reductions in lung function at age 10 years for post‐bronchodilator FEV_1_ in both sensitized (−0.25, 95% CI: −0.55 to 0.05 *z*‐score units, *p* = .108, *n* = 285) and non‐sensitized (−0.12, 95% CI: −0.26 to 0.02 *z*‐score units, *p* = .081, *n* = 1179) children. Similar patterns were observed for FEV_1_/FVC, FVC, and FEF_25–75_. Associations were weaker and less consistent at age 6 years.

**Conclusion:**

A two‐hit interaction between food sensitization and respiratory infection during infancy on subsequent lung function was not confirmed. Early‐life lower respiratory infection was however associated with modest reductions in lung function at age 10 years, regardless of sensitization status.

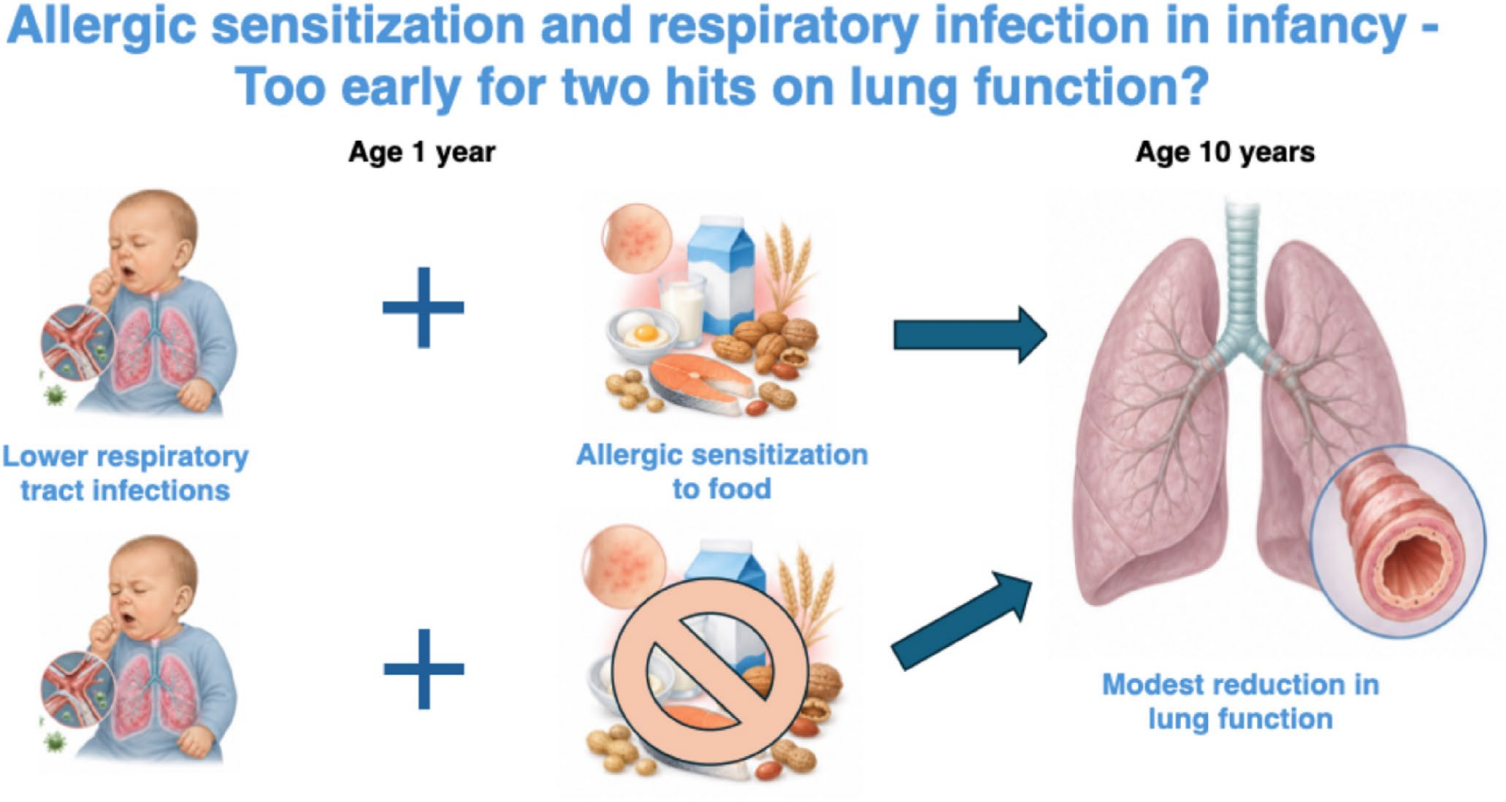

## Graphical abstract

Lower respiratory tract infections during infancy were associated with modest impairment of lung function by age 10 years. This was observed in both children with and without sensitisation to food allergens at age 1 year (Images created with support of Chat GPT).

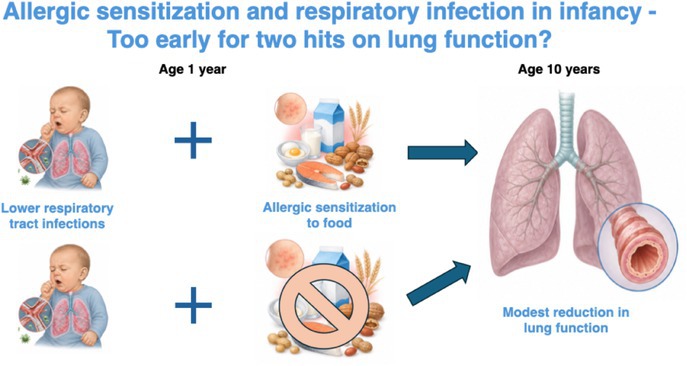

**Abbreviations**
BDR: bronchodilator responsiveness
DAG: directed acyclic graph
FEF_25–75_
: forced expiratory flow between 25% and 75% of FVC
FEV_1_
: forced expiratory volume in 1 s
FVC: forced vital capacity
HRV: human rhinovirus
RSV: respiratory syncytial virus

**Key message**

Our study investigated for a relationship between combined food sensitization and respiratory infections during infancy on lung function outcomes at ages 6 and 10 years. Our results do not confirm a two‐hit effect between allergic sensitization to food allergens and respiratory infection during infancy on childhood lung function. Children with lower respiratory infections during the first year of life had modest reductions in lung function at age 10 years, irrespective of their sensitization status to food allergens. There was, however, no evidence for a statistical interaction.

## 1 INTRODUCTION

Lung function development is complex and able to be influenced by multiple adverse influences in prenatal and early life periods as well as during childhood.^1^, ^2^, ^3^, ^4^ Failure to attain optimal lung function in childhood may track towards lower lung function in adulthood and potentially result in chronic lung disease as part of the aging process.^5^, ^6^, ^7^ Early‐life respiratory infection and allergic sensitization are both common causes of wheezing in childhood and are believed to be important in influencing lung growth.^3^, ^4^, ^8^, ^9^, ^10^ In various birth cohort studies, different lung function trajectories have been identified as negative sequalae of various early‐life factors in the critical and vulnerable period of early lung development.^2^, ^3^, ^11^

The “two hit” hypothesis argues that early‐life allergic sensitization and respiratory infection interact and are associated with an increase in adverse respiratory outcomes.^12^, ^13^, ^14^, ^15^, ^16^, ^17^ Previous studies have already observed that both of these factors also act independently to influence respiratory health.^4^, ^10^, ^18^, ^19^, ^20^ Early‐life allergic sensitization to various aero and food allergens has been associated with the development of persistent wheeze, asthma, and lung function impairment both in childhood and into early adulthood.^20^, ^21^, ^22^, ^23^, ^24^ Bronchiolitis is the most common lower respiratory tract infection in children below age 2 years.^25^ It is most commonly viral in origin with Respiratory Syncytial Virus (RSV) the predominant pathogen in infancy; however, many other pathogens including influenza and human rhinovirus (HRV) are also believed important.^4^, ^9^, ^26^ Early‐life respiratory infections, particularly when severe, recurrent or associated with wheezing, may be associated with subsequent asthma, impaired lung function and bronchial hyperresponsiveness.^4^, ^10^, ^26^, ^27^, ^28^, ^29^, ^30^

Most studies investigating combined effects of early‐life allergic sensitization and early‐life respiratory infection have assessed these two factors at age 2 years or later on subsequent asthma outcomes.^1^, ^13^, ^31^ Those that considered sensitization by the age of 2 years had observed greater associations with asthma risk.^17^, ^32^ A previous study that assessed the relationship between respiratory infection and sensitization by the age of 2 years and adult lung function found increased risks for reduced lung function in those with sensitization compared to those who were not sensitized.^13^ It is, however, not known whether allergic sensitization and respiratory infections can interact during the critical lung developmental window of the first year of life and potentially have stronger relationships with subsequent childhood lung function.

## 2 OBJECTIVES

In a large population‐based longitudinal study, we aimed to investigate whether allergic sensitization to common food allergens and respiratory infection during infancy interact to negatively impact childhood lung function in a multiplicative manner.

## 3 METHODS

### 3.1 Study cohort and design

HealthNuts recruited 5276 infants at the age of 12 months (range 11–15 months) between 2007 and 2011 from 138 council‐run immunization centers across Melbourne, Australia. It is a single‐centre, multi‐wave study that was designed to assess prevalence and determinants of allergy, and to date has followed these children up to age 10 years. The HealthNuts cohort profile has been described and published previously.^33^

Ethics approval for HealthNuts was provided by the Victorian Department of Human Services (reference no. 10/07), the Office for Children (reference no. CDF/07/492), and the Royal Children's Hospital Human Research Ethics Committee (reference nos. 27047 and 32294). Parents or guardians provided informed consent on behalf of their children.

### 3.2 Data collection

Methods utilized in HealthNuts have been published in detail previously.^33^, ^34^, ^35^, ^36^

During Wave 1 at age 1 year (*n* = 5276), parent‐completed questionnaires and skin‐prick testing to egg, peanut, sesame, and either cow's milk or shellfish were performed. Those with detectable wheals (at least 1 mm) subsequently underwent oral food challenges to determine the presence of clinical food allergy. Parent or guardian administered questionnaires also sought responses to questions in relation to sex at birth, gestation, ethnicity, pet ownership (cat or dog), exposure to passive smoke, parental allergic disease (hay fever, eczema, asthma, or food allergy), and breastfeeding. Socioeconomic status was determined using the Index of Relative Socioeconomic Disadvantage (IRSD) provided by the Australian Bureau of Statistics. This index was used as a proxy for socioeconomic status and classified into quintiles of the Australian Socio‐economic Indexes for Areas (SEIFA). Questionnaires also included information on episodes of respiratory infection.

During Wave 2 at age 4 years (*n* = 4291), caregivers completed questionnaires. Food challenges were repeated in children previously identified as food allergic to determine resolution and those who developed new food reactions to diagnose food allergy or tolerance.

During Wave 3 at age 6 years (*n* = 3233), all children were invited for clinical assessment, including lung function, physical measurements of height, weight and waist circumference, skin‐prick testing to foods and aeroallergens, and food challenge testing if food sensitization was present. Detailed questionnaires were also completed by caregivers.

At age 10 years (Wave 4), questionnaires, allergy status and lung function were reassessed as described for 6 years.

### 3.3 Exposures

Allergic sensitization was assessed using skin prick testing (SPT) and blood tests for specific IgE which were undertaken at age 1 year to egg, peanut, sesame and either cow's milk or shellfish (ALK‐Abelló, Madrid, Spain) with a single use lancet (Stallergenes Greer, Antony, France). Infants who had detectable wheal reactions of at least 2 mm to at least one of the first 3 allergens (*n* = 1089) were offered oral food challenge testing in the clinic, along with expanded allergic sensitization testing to milk, egg, peanut, wheat, sesame, cashew, almond, and hazelnut. House dust mite (HDM) sensitization was assessed in a subset of the cohort (*n* = 1116). Wheal size was calculated as the mean of the longest and perpendicular diameters.^37^ Positive (10 mg/mL histamine) and negative (saline) control were performed.^38^ All SPTs were conducted by an allergy researcher on infants' backs and read after 15–20 min. Only children with positive community‐based SPTs were included in the current study for analysis.

Respiratory infection was restricted to lower respiratory tract symptoms and assessed through responses to questionnaires, completed by parents or guardians when infants were approximately 12 months of age. Questions asked included “Has your child ever had bronchiolitis?”, “Was your child hospitalised for bronchiolitis?”, and “Has your child ever had antibiotics?”—if the answer to the last question was “yes” then a reason for the use of antibiotics was requested. A respiratory infection was considered to have been present if there was a positive response to (1) either of the first two questions (bronchiolitis), or (2) to the third question, if the reason for antibiotic use was to treat a chest infection. Respiratory infection was dichotomized and treated as a binary variable.

### 3.4 Outcomes

Lung function was assessed at ages 6 and 10 years with the EasyOne Spirometer (ndd Medical Technologies, Andover, MA, USA). Pre‐bronchodilator and post‐bronchodilator (10 min after administering four puffs of 100 μg salbutamol) spirometry were performed according to American Thoracic Society and European Respiratory Society (ATS/ERS) guidelines. Acceptability of trials was in accordance with ATS/ERS guidelines and only acceptable trials with quality scores A–C, representing at least two acceptable and repeatable measurements, were included in the analysis.^34^ Bronchodilator responsiveness was defined as an increase in FEV_1_ or FVC of at least 200 mL, and at least 12% relative to pre‐bronchodilator values. We retained the 2005 ATS/ERS criteria for the assessment of BDR rather than the updated 2021 criteria of >10% change of predicted values for FEV_1_ or FVC.^39^

Reference values were derived from the Global Lung Function Initiative (GLI) reference equations that have been validated in an Australasian population. Raw values were transformed to z scores for FEV_1_, FVC, and FEV_1_/FVC using the GLI Tool Software developed by the GLI Task Force Group (ERS) and standardized for age, sex, and height. ß coefficients of z scores and changes in lung function were reported when these were used as outcome variables.

### 3.5 Statistical methods

Characteristics of the children and parents have been described using percentages, means, and confidence intervals where appropriate. Multivariate linear regression and statistical interaction analyses were performed to assess relationships between allergic sensitization and respiratory infection by the age of 1 year and lung function at ages 6 and 10 years. In particular, we considered the association between respiratory infection and lung function separately between sensitized and non‐sensitized groups. We reported stratified results even where there were no findings of interaction between sensitization and respiratory infection. For each stratum, *z*‐scores and absolute lung function values were assessed at both age timepoints, including 95% confidence intervals and p‐values for changes in FEV_1_, FVC, FEV_1_/FVC, and FEF_25–75_.

A directed acyclic graph (DAG) was created to determine a minimum set of confounders (Appendices S1 and S2). Potential confounders included sex, height, gestational age, breast feeding, parental allergy, day care attendance, older siblings, maternal education level, socio‐economic status, environmental tobacco smoke, and pet ownership. When all were included, there was a sizeable proportion of missing data, and we therefore elected to undertake analysis, excluding day care use and maternal education, which had the greatest missing data. This resulted in 2194 children able to be included for analysis at age 6 years with acceptable pre‐bronchodilator spirometry and 1960 children with acceptable post‐bronchodilator spirometry. At age 10 years, 1622 children were included for analysis who had acceptable pre‐bronchodilator and 1464 with acceptable post‐bronchodilator spirometry. A sensitivity analysis was undertaken with all confounding factors included and a subsequent increase in missing data. In a second sensitivity analysis, we also assessed for an interaction between allergic sensitization and respiratory infection at both ages 6 and 10 years, across sub‐groups of those with and without asthma. In the final sensitivity analysis, we assessed for interactions between HDM sensitization and respiratory infection. Models were adjusted for potential confounding variables, and all analyses were conducted using Stata version 18 (2023 StataCorp, College Station, Texas).

## 4 RESULTS

**FIGURE 1:**
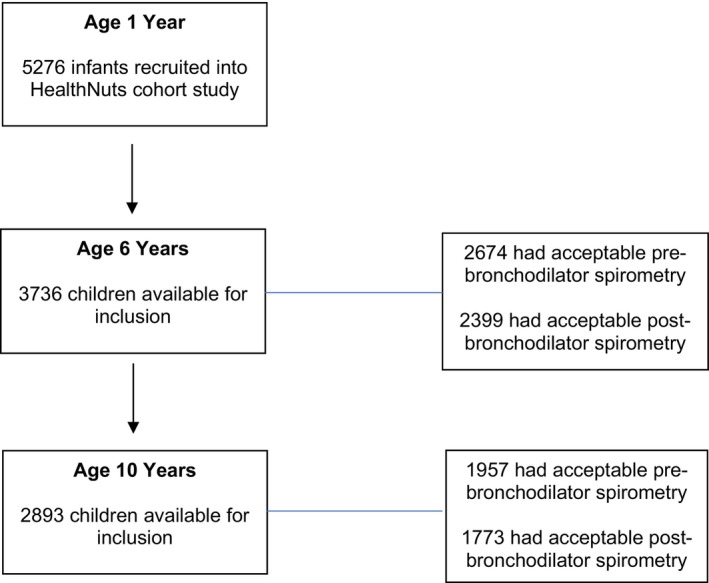
HealthNuts Study population flow diagram up to age 10 years.

At the baseline age of 1 year, there were 5276 infants recruited; however, through progressive attrition due to loss to follow up or missing data, 3736 and 2893 infants continued in HealthNuts at ages 6 and 10 years, respectively (Figure 1). Those lost to follow up were more likely to be of a culturally or linguistically diverse background, reside in lower socioeconomic areas, and be exposed to passive smoke (Table 1). They were also less likely to have a family history of allergies.

**TABLE 1:** HealthNuts Study population characteristics at recruitment and up to 10 years.

Enrolled	At 1 year	Age 6 years	Age 10 years
	(*n* = 5276)	(*n* = 3736)	(*n* = 2893)
Sex, *n* (%)			
Male	2665/5244 (50.8)	1921/3736 (51.4)	1481/2893 (51.2)
Female	2579/5244 (49.2)	1815/3736 (48.6)	1412/2893 (48.8)
Age (y), mean +/− SD	1.05 (0.06)	6.30 (0.4)	10.38 (0.32)
Height (cm), mean +/− SD	—	119.23 (5.61)	143.44 (6.87)
Missing		669	754
Ethnicity (Caucasian), *n* (%)		2543/3067 (82.9)	1822/2049 (88.9)
Parental allergy, *n* (%)	3661/5276 (69.4)	2670/3736 (72.2)	2100/2893 (72.3)
Siblings, *n* (%)	2647/5251 (50.4)		
Pet ownership, *n* (%)	2165/2324 (93.2)	1852/3609 (51.3)	2024/3227 (62.7)
Maternal education (Tertiary), *n* (%)*at age 4 years		2319/2690 (86.2)	
Passive smoke exposure (current), *n* (%)	1127/5234 (21.5)	399/3736 (10.7)	296/2893 (10.8)
Low SES status (ISRD cat 5), *n* (%)	1028/5261 (19.5)	688/3736 (18.4)	609/2893 (21.1)
Premature gestation (=<36 weeks), *n* (%)	151/4750 (3.2)	208/3365 (6.2)	185/3038 (6.1)
Exclusive breast feeding 4 months, *n* (%)	3919/5018 (78.1)	2781/3428 (81.1)	2501/3074 (81.4)
Lung function			
Pre‐BD acceptable spirometry *n* (%)	—	2674/3067 (87.2)	1957/2136 (91.6)
*z*‐score FEV_1_ (mean (SD); CI)		−0.18 (1.02); −4.44, 7.61	−0.12 (0.99); −5.00, 3.69
Post BD Acceptable spirometry *n* (%)	—	2399/2812 (85.3)	1773/1922 (92.2)
*z*‐score FEV_1_ (mean, SD, CI)		0.20 (1.07); −4.37, 3.77	0.25 (1.01); −4.33, 4.21
Bronchodilator responsiveness (BDR), *n* (%)		287/2399 (11.92)	154/1773 (8.67)
SPT positivity (2mm+) community test Year 1	895/5137 (17.42)	—	—
Respiratory infection year 1, *n* (%)	1106/5258 (20.90)	—	—

Demographics of children in the HealthNuts cohort across all 3 age groups and summary statistics of exposure and outcome data are as shown (Table 1). Proportions of children who were male were 50.8% at age 1 year, 51.4% at age 6 years, and 51.2% at age 10 years. There were 895 children (17.4%) with sensitization to food allergens and 1106 (20.9%) who reported a history of respiratory infection in the first year of life (Table 1). In the subset of children who were assessed for allergic sensitization to HDM allergen (*n* = 1116), 90 were positive on skin prick testing (8.1%).

There were 2674 and 1957 children at ages 6 and 10 years, respectively, who had acceptable lung function data and who were able to be included in the current study. BDR positivity at ages 6 and 10 years was 11.96% and 8.67%, respectively (Table 2).

**TABLE 2:** HealthNuts cohort with bronchodilator responsiveness (BDR) positivity at ages 6 and 10 years.

	Age 6 years (*n* = 2399)	Age 10 years (*n* = 1773)
BDR positivity for FEV1	215 (8.96%)	147 (8.28%)
BDR positivity for FVC	180 (7.50%)	21 (1.18%)
BDR positivity for both FEV1 and FVC	108 (4.50%)	14 (0.79%)
Any BDR positivity	287 (11.96%)	154 (8.67%)

## 5 MAIN FINDINGS

### 5.1 Age 6 years

There was no evidence for an interaction between respiratory infection and food sensitization in infancy on pre‐and post‐bronchodilator lung function, measured at age 6 years (Tables 3 and 4). In both sensitized and non‐sensitized children, children with respiratory infections in infancy had modest reductions in FEV_1_, FVC, and FEV_1_/FVC z‐scores, compared to children without respiratory infections; however, 95% CI were wide and included the null. Individuals with respiratory infections during the first year of life also had a greater reduction in pre‐ and post‐bronchodilator FEF_25–75_ compared with those without infections; however, there was no material difference observed by sensitisation status. Differences in absolute lung function parameters paralleled trends for z‐scores, with the greatest reductions observed for FEF_25‐75_ (Tables S1 and S2). Sensitivity analysis did not identify an interaction between allergic sensitization and respiratory infection in children across sub‐groups of those with and without asthma (Table S2B). In the subset of children who had SPT to HDM allergen, there was also no evidence for an interaction with respiratory infection (Table S5).

**TABLE 3:** Association between respiratory infection at age 1 year and PRE‐Bronchodilator lung function outcomes (*z*‐scores) at age 6 years, stratified by food allergen sensitization at age 1 year.

	Sensitized (*n* = 409)		Not sensitized (*n* = 1785)		Interaction (*n* = 2194)
	Coefficient^a^ (95%CI)	*p* Value	Coefficient^a^ (95% CI)	*p* Value	*p* Value
*Z*‐score FEV_1_	−0.05 (−0.29, 0.19)	*p* = .686	−0.04 (−0.16, 0.07)	*p* = .453	*p* = .950
*Z*‐score FVC	−0.05 (−0.31, 0.21)	*p* = .715	−0.01 (−0.13, 0.11)	*p* = .825	*p* = .947
*Z*‐score FEV_1_/FVC	−0.14 (−0.41, 0.13)	*p* = .308	−0.06 (−0.19, 0.07)	*p* = .343	*p* = .422
*Z*‐score FEF_25‐75_	−0.22 (−0.45, 0.01)	*p* = .061	−0.13 (−0.24, −0.02)	***p* = .023**	*p* = .441

*Note:* The bold values indicate statistical significance (*p* <.05)

^a^ Adjusted for gestation, breast feeding, siblings, passive smoke exposure, socioeconomic class, family allergy, and maternal ethnicity.

**TABLE 4:** Association between respiratory infection at age 1 year and POST‐bronchodilator lung function outcomes (*z*‐scores) at age 6 years, stratified by food allergen sensitization at age 1 year.

	Sensitized (*n* = 361)		Not sensitized (*n* = 1599)		Interaction (*n* = 1960)
	Coefficient^a^ (95% CI)	*p* Value	Coefficient^a^ (95% CI)	*p* Value	*p* Value
*Z*‐score FEV_1_	0.04 (−0.23, 0.31)	*p* = .769	−0.02 (−0.15, 0.11)	*p* = .763	*p* = .808
*Z*‐score FVC	0.02 (−0.26, 0.31)	*p* = .871	−0.01 (−0.14, 0.12)	*p* = .888	*p* = .918
*Z*‐score FEV_1_/FVC	0.00 (−0.29, 0.28)	*p* = .989	−0.03 (−0.16, 0.09)	*p* = .604	*p* = .954
*Z*‐score FEF_25–75_	−0.11 (−0.39, 0.16)	*p* = .427	−0.16 (−0.29, −0.03)	***p* = .019**	*p* = .864

*Note:* The bold values indicate statistical significance (*p* <.05)

^a^ Adjusted for gestation, breast feeding, siblings, passive smoke exposure, socioeconomic class, family allergy, and maternal ethnicity.

### 5.2 Age 10 years

Similar to observations at age 6 years, there was no evidence for a statistical interaction between respiratory infection and food sensitization in infancy on pre‐and post‐bronchodilator lung function measured at age 10 years for FEV_1_, FVC, FEV_1_/FVC, and FEF_25–75_. Comparison to findings at age 6 years, however, respiratory infection in infancy was associated with greater reductions in lung function, for most parameters except FVC, parameters at age 10 years among both those who were sensitized and those non‐sensitized (Tables 5 and 6).

**TABLE 5:** Association between respiratory infection at age 1 year and PRE‐bronchodilator lung function outcomes (*z*‐scores) at age 10 years, stratified by food allergen sensitization at age 1 year.

	Sensitized (*n* = 310)		Not sensitized (*n* = 1312)		Interaction (*n* = 1622)
	Coefficient^a^ (95% CI)	*p* Value	Coefficient^a^ (95% CI)	*p* Value	*p* Value
*Z*‐score FEV_1_	−0.26 (−0.56, 0.03)	*p* = .078	−0.15 (−0.28, −0.02)	***p* = .022**	*p* = .487
*Z*‐score FVC	−0.13 (−0.40, 0.14)	*p* = .348	−0.07 (−0.19, 0.06)	*p* = .300	*p* = .658
*Z*‐score FEV_1_/FVC	−0.19 (−0.46, 0.08)	*p* = .167	−0.14 (−0.27, −0.02)	***p* = .022**	*p* = .795
*Z*‐score FEF_25–75_	−0.33 (−0.62, −0.04)	***p* = .026**	−0.19 (−0.32, −0.06)	***p* = .005**	*p* = .456

*Note:* The bold values indicate statistical significance (*p* <.05)

^a^ Adjusted for gestation, breast feeding, siblings, passive smoke exposure, socioeconomic class, family allergy, and maternal ethnicity.

**TABLE 6:** Association between respiratory infection at age 1 year and POST‐bronchodilator lung function outcomes (*z*‐scores) at age 10 years, stratified by food allergen sensitization at age 1 year.

	Sensitized (*n* = 285)		Not sensitized (*n* = 1179)		Interaction (*n* = 1464)
	Coefficient^a^ (95% CI)	*p* Value	Coefficient^a^ (95% CI)	*p* Value	*p* Value
*Z*‐score FEV_1_	−0.25 (−0.55, 0.05)	*p* = .108	−0.12 (−0.26, 0.02)	*p* = .081	*p* = .437
*Z*‐score FVC	−0.18 (−0.46, 0.10)	*p* = .200	−0.04 (−0.17, 0.08)	*p* = .501	*p* = .324
*Z*‐score FEV_1_/FVC	−0.10 (−0.35, 0.15)	*p* = .442	−0.14 (−0.26, −0.02)	***p* = .025**	*p* = .622
*Z*‐score FEF_25–75_	−0.27 (−0.56, 0.02)	*p* = .064	−0.20 (−0.34, −0.05)	***p* = .007**	*p* = .782

*Note:* The bold values indicate statistical significance (*p* <.05)

^a^ Adjusted for gestation, breast feeding, siblings, passive smoke exposure, socioeconomic class, family allergy, and maternal ethnicity.

Whilst numerically greater reductions in lung function parameters were observed in food sensitized compared to non‐sensitized children, confidence intervals were wide and overlapping. In children with allergic sensitization and respiratory infections during infancy, pre‐bronchodilator FEV_1_ reductions (−0.26, 95% CI: −0.56, 0.03, *z*‐score units, (*p* = .078)) were larger in comparison to those who had respiratory infections but who were not sensitized (−0.15, 95% CI: −0.28, −0.02, *z*‐score units, (*p* = .022)) however there was no evidence for an interaction (*p*_interaction_ *p* = .487). Similar findings were observed for pre bronchodilator FVC in the sensitized group (−0.13, 95% CI: −0.40, 0.14, *z*‐score units, (*p* = .348)) in comparison to the non‐sensitized group (−0.07, 95% CI: −0.19, 0.06, *z*‐score units, (*p* = .300)).

For post‐bronchodilator lung function, similar patterns were observed for parameters of FEV_1_, FVC, and FEF_25–75_ (Table 6).

FEV_1_/FVC also showed small reductions but with no material difference between the sensitized and non‐sensitized groups across both pre and post bronchodilator spirometry. Changes in absolute lung function parameter values paralleled trends observed for z‐scores (Tables S3 and S4). Sensitivity analysis, again, did not identify an interaction between allergic sensitization and respiratory infection across sub‐groups of those with and without asthma (Table S4B). In the subset of children who had SPT to HDM allergen, there was also no evidence for an interaction with respiratory infection (Table S5B).

## 6 DISCUSSION

To our knowledge, this is the first study that has examined for a potential interaction between allergic sensitization to food allergens and lower respiratory tract infection in the first year of life, and subsequent lung function. There was no evidence of a statistical interaction between sensitization to food allergens and respiratory infection in infancy on subsequent lung function up to age 10 years. Although modestly greater reductions in lung function were observed at age 10 years among sensitized children with respiratory infection compared with non‐sensitized children, confidence intervals were wide and overlapping.

It is believed that impediments along the immune maturation pathway may predispose some children to early‐life sensitization.^22^ Food sensitization usually peaks in early‐life, generally within the first 1 to 2 years; however, there may be potential for developing tolerance to the sensitized food. Conversely, aeroallergen sensitization has a tendency to increase over time and is not usually associated with spontaneous resolution.^40^ Whilst in some there may be cross‐reactivity and overlap through IgE, sensitization to food allergens may be due to pathways distinct from these and include cell‐mediated mechanisms.^41^ Early sensitization to food allergens by age 1 year, however, may identify a cohort that may develop sensitization to further allergens over time.^40^, ^42^, ^43^ Food allergy in infancy is considered an important factor that has been associated with asthma outcomes.^44^ Food allergy has also been associated with lung function impairment, with reduced FEV_1_, FVC, and FEF_25–75_ that may be independent of an asthma diagnosis, suggesting that there may be immune or inflammatory pathways that are distinct from those involved with asthma development.^34^, ^45^, ^46^ Whilst food sensitization may be a critical factor for respiratory morbidity on its own, an interplay of sensitization with genetic, immunological, and other factors may also contribute to airway remodeling that eventually results in asthma, airway hyperresponsiveness, and lung function impairment.^16^, ^27^, ^47^, ^48^

Infancy represents a vulnerable period of lung development with narrowed peripheral airways. Severe respiratory infection, frequently associated with RSV and presenting as bronchiolitis during this time, can interfere with normal alveolarization and prevent the attainment of optimal lung function.^49^ Innate and adaptive immune responses are nevertheless also believed to play important roles in the development of bronchiolitis.^50^ Prematurity has been recognized to be an important factor with early‐life RSV infections, which are more frequent and severe in affected children.^27^, ^29^ Additionally, these children are more likely to develop persistent wheeze and show trends towards lower lung function.^27^, ^29^ Respiratory infection is known to be associated with inflammation, which in early life can play an important role in inducing airway remodeling.^16^, ^27^, ^47^, ^48^ Despite this, the mechanisms linking early‐life respiratory infection and lung function impairment remain unclear, as only some affected children will progress to developing persistent wheeze, asthma, or abnormal lung function.^27^

Early‐life respiratory infection can also be associated with the subsequent development of allergic sensitization.^9^, ^51^, ^52^ Sensitization in turn may be associated with adverse responses to viral infections, and in combination, these exposures may be associated with an increase in adverse respiratory sequelae.^9^ Potential mechanisms for an association between early life sensitization and respiratory infection include defects of the airway epithelial barrier, impaired innate immune responses, and enhanced inflammatory mediator release.^53^, ^54^ Coinfections as well as greater severity and more frequent respiratory infections in early life have been proposed as potential reasons for the observed increase in respiratory morbidity.^9^, ^11^, ^12^, ^14^, ^16^, ^26^, ^55^, ^56^, ^57^, ^58^

Interactions between sensitization and respiratory infection in early childhood and asthma development have not been conclusively established.^31^, ^59^ Studies that have identified associations between both these early‐life factors and asthma have noted sensitization and respiratory infection by the age of 2 years, considered to be a critical timepoint for vulnerability in lung development.^15^, ^17^, ^32^, ^60^ An increasing frequency of respiratory infection has also been associated with a greater likelihood for subsequent asthma.^31^, ^59^, ^61^ Where these factors have been identified in later years, an interaction effect has not been observed.^18^, ^61^, ^62^

To date only one study has investigated the interactive effects of early‐life sensitization and respiratory infection on lung function outcomes.^13^ This birth cohort study found evidence for interactions between these early‐life exposures on lung function at ages 18 and 25 years. In addition, there were also differential relationships with lung function dependent upon whether sensitization was present or absent in early‐life. In those with allergic sensitization by age 2 years, respiratory infection was associated with reduced lung function up to the age of 25 years; however, early‐life respiratory infections in the absence of allergic sensitization were associated with an improvement in lung function. This suggests that there may be protective effects of early life respiratory infections, potentially through favorable impacts on the developing immune system.^13^, ^16^ These findings differ from our results which showed no such protective effect of early‐life respiratory infection in those without allergic sensitization.

Strengths of this study include the use of a large population‐based cohort with high participation rates and a long follow up time period that supports generalizability to a wider community population. Multiple covariates were able to be included to address confounding and validated questionnaires were used in our analysis to document exposure, which increases the robustness of the outcome spirometry data. There are notable weaknesses, predominantly that of a limited number of food allergens and the absence of aeroallergens being assessed for the presence of sensitization in infancy. This may have contributed to misclassification bias and limited our ability to detect an interaction with early life respiratory infection. Respiratory infection was also based on parental or guardian reports of doctor diagnosed bronchiolitis (with or without hospitalization) and in those who were treated with antibiotics for chest infection but without microbiological pathogen confirmation. This may also have introduced misclassification bias and potentially limited the number of children able to be included to those with more severe and only lower respiratory tract infective symptoms. There was also a differential loss to follow up, particularly of those from lower socioeconomic class, cultural and linguistically diverse background, and exposure to passive smoke, which may have been associated with selection bias. In addition, exclusion of subjects due to lack of reproducible spirometry, as well as being unable to adjust for potential confounding due to day care attendance and maternal education, may have limited both the size of the cohort analyzed as well as our ability to draw strong conclusions on the association.

Our findings have public health interest and relevance. Although there was no synergistic two‐hit effect observed between sensitization to food allergens and lower respiratory infection in infancy on subsequent lung function to age 10 years, our results support the importance of maintaining early‐life respiratory health. Minimizing exposure to lower respiratory infections, irrespective of underlying sensitization status, may help infants and young children in achieving optimal lung function.

In conclusion, we found no evidence supporting a two‐hit interaction between food sensitization and lower respiratory infection in infancy on lung function up to age 10 years. Early life lower respiratory infection was, however, associated with modest reductions in lung function in childhood. Further research is needed to elucidate mechanisms that link early life respiratory infections to lung function trajectories and define whether specific subgroups may be more susceptible to adverse outcomes.

## AUTHOR CONTRIBUTIONS

**Caroline Lodge:** Validation; visualization; writing – review and editing; project administration; supervision; resources. **Vikas Wadhwa:** Conceptualization; investigation; writing – original draft; methodology; validation; visualization; writing – review and editing; formal analysis; project administration; data curation. **Mimi L. K. Tang:** Writing – review and editing; visualization; validation. **Katrina J. Allen:** Writing – review and editing; visualization. **Jennifer J. Koplin:** Visualization; validation; writing – review and editing; supervision. **Adrian J. Lowe:** Validation; visualization; writing – review and editing. **Shyamali C. Dharmage:** Validation; visualization; writing – review and editing; project administration; supervision; resources. **Melissa Russell:** Conceptualization; methodology; validation; visualization; writing – review and editing; project administration; supervision; resources. **Rachel L. Peters:** Conceptualization; investigation; methodology; validation; visualization; writing – review and editing; formal analysis; supervision; resources.

## FUNDING INFORMATION

This work was supported by funding from the NHMRC of Australia, the Ilhan Food Allergy Foundation, AnaphylaxiStop, the Charles and Sylvia Viertel Medical Research Foundation and the Victorian Government’s Operational Infrastructure Support Program.

## CONFLICT OF INTEREST STATEMENT

AJL has received an investigator‐initiated grant from GlaxoSmithKline (GSK) and Sanofi Regeneron for unrelated research. He has received investigational product (EpiCeram TM) free of charge from Primus Pharmaceuticals for use in unrelated research. JJK and RLP received a research award from the Stallergenes Greer Foundation, paid to their institution, unrelated to the current manuscript. All authors declare that they have no relevant conflicts of interest.

## Supporting information


Data S1.

## Data Availability

The data that support the findings of this study are available from the corresponding author upon reasonable request.
