# Supplementary material for: Allergic sensitization and respiratory infection in infancy—Too early for two‐hits on lung function?
Source: Pediatr Allergy Immunol. 2026 Jul 21;37(7):e70427. doi: 10.1111/pai.70427 (PMC13389556; doi:10.1111/pai.70427)
Supplement: Supplementary file 1 — Data S1. [file PAI-37-e70427-s001.docx]

Appendix 1:

**Detailed Directed Acyclic Graph of relationships between respiratory infection and food allergen sensitisation in infancy, other potential variables, and outcome of early childhood lung function. (Image produced using DAGitty software V2.3 (**[**55**](#_ENREF_55)**); See Appendix 2 for DAGitty Model Code)**

~~
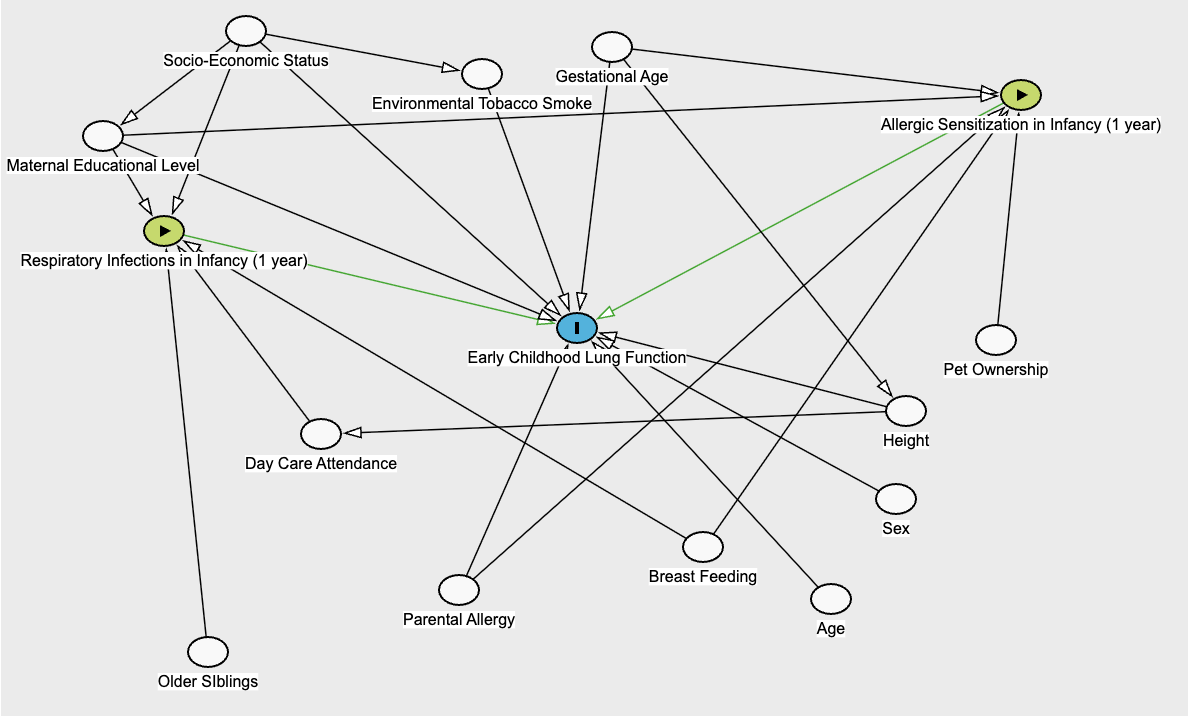
~~

Appendix 2:

Daggity Code (Daggity 3.1)

dag {

bb="0,0,1,1"

"Allergic Sensitization in Infancy (1 year)" [exposure,pos="0.853,0.130"]

"Breast Feeding" [adjusted,pos="0.587,0.651"]

"Day Care Attendance" [adjusted,pos="0.267,0.521"]

"Early Childhood Lung Function" [outcome,pos="0.481,0.399"]

"Environmental Tobacco Smoke" [adjusted,pos="0.402,0.106"]

"Gestational Age" [adjusted,pos="0.510,0.075"]

"Maternal Educational Level" [adjusted,pos="0.084,0.177"]

"Older SIblings" [adjusted,pos="0.172,0.772"]

"Parental Allergy" [adjusted,pos="0.382,0.700"]

"Pet Ownership" [adjusted,pos="0.832,0.412"]

"Respiratory Infections in Infancy (1 year)" [exposure,pos="0.135,0.287"]

"Socio-Economic Status" [adjusted,pos="0.204,0.056"]

Age [adjusted,pos="0.694,0.711"]

Height [adjusted,pos="0.757,0.494"]

Sex [adjusted,pos="0.749,0.596"]

"Allergic Sensitization in Infancy (1 year)" -> "Early Childhood Lung Function"

"Breast Feeding" -> "Allergic Sensitization in Infancy (1 year)"

"Breast Feeding" -> "Respiratory Infections in Infancy (1 year)"

"Day Care Attendance" -> "Respiratory Infections in Infancy (1 year)"

"Environmental Tobacco Smoke" -> "Early Childhood Lung Function"

"Gestational Age" -> "Allergic Sensitization in Infancy (1 year)"

"Gestational Age" -> "Early Childhood Lung Function"

"Gestational Age" -> Height

"Maternal Educational Level" -> "Allergic Sensitization in Infancy (1 year)"

"Maternal Educational Level" -> "Early Childhood Lung Function"

"Maternal Educational Level" -> "Respiratory Infections in Infancy (1 year)"

"Older SIblings" -> "Respiratory Infections in Infancy (1 year)"

"Parental Allergy" -> "Allergic Sensitization in Infancy (1 year)"

"Parental Allergy" -> "Early Childhood Lung Function"

"Pet Ownership" -> "Allergic Sensitization in Infancy (1 year)"

"Respiratory Infections in Infancy (1 year)" -> "Early Childhood Lung Function"

"Socio-Economic Status" -> "Early Childhood Lung Function"

"Socio-Economic Status" -> "Environmental Tobacco Smoke"

"Socio-Economic Status" -> "Maternal Educational Level"

"Socio-Economic Status" -> "Respiratory Infections in Infancy (1 year)"

Age -> "Early Childhood Lung Function"

Height -> "Day Care Attendance"

Height -> "Early Childhood Lung Function"

Sex -> "Early Childhood Lung Function"

**Supplementary Table 1:** **Association between Respiratory Infection at age 1 year and PRE- Bronchodilator lung function outcomes (absolute change) at age 6 years, stratified by food allergen sensitization at age 1 year**

|  | **Sensitized**  (n=409) | | **Not Sensitized**  (n=1785) | | **Interaction**  (n=2194) |
| --- | --- | --- | --- | --- | --- |
|  | **Coefficient (95%CI)** | **p-value** | **Coefficient (95%CI)** | **p-value** | **p-value** |
| **FEV_1 (mls)_** | -2.83 (-41.52, 35.85) | p=0.886 | -6.25 (-25.15, 12.66) | p=0.517 | p=0.951 |
| **FVC (mls)** | 7.36 (-37.27, 51.98) | p=0.746 | -0.64 (-22.73, 21.46) | p=0.955 | p=0.799 |
| **FEV_1_/FVC (%)** | -0.80 (-2.34, 0.74) | p=0.306 | -0.37 (-1.08, 0.34) | p=0.304 | p=0.380 |
| **FEF_25-75_ (mls/sec)** | -92.39 (-189.22, 4.43) | p=0.061 | -51.10 (-98.40, -3.79) | **p=0.034** | p=0.338 |

* Adjusted for age, height, gestation, breast feeding, siblings, gender, passive smoke exposure, socioeconomic class, family allergy and maternal ethnicity

**Supplementary Table 2:** **Association between Respiratory Infection at age 1 year and POST- Bronchodilator lung function outcomes (absolute change) at age 6 years, stratified by food allergen sensitization at age 1 year**

|  | **Sensitized**  (n=361) | | **Not Sensitized**  (n=1599) | | **Interaction**  (n=1960) |
| --- | --- | --- | --- | --- | --- |
|  | **Coefficient (95%CI)** | **p-value** | **Coefficient (95%CI)** | **p-value** | **p-value** |
| **FEV_1 (mls)_** | 8.39 (-33.97, 50.76) | p=0.697 | -0.40 (-21.36, 20.56) | p=0.970 | p=0.939 |
| **FVC (mls)** | 13.53 (-34.41, 61.47) | p=0.579 | 0.29 (-23.34, 23.93) | p=0.981 | p=0.801 |
| **FEV_1_/FVC (%)** | -0.13 (-1.69, 1.43) | p=0.873 | -0.09 (-0.80, 0.61) | p=0.792 | p=0.891 |
| **FEF_25-75_ (mls/sec)** | -64.25 (-180.56, 52.07) | p=0.278 | -61.24 (-117.84, -4.63) | **p=0.034** | p=0.904 |

* Adjusted for age, height, gestation, breast feeding, siblings, gender, passive smoke exposure, socioeconomic class, family allergy and maternal ethnicity

**Supplementary Table 2B:** **Association between Respiratory Infection at age 1 year and POST- Bronchodilator lung function outcomes (z- scores) at age 6 years, in relation to history of asthma and stratified by food allergen sensitization at age 1 year**

|  | **Sensitized**  (n=361) | | **Not Sensitized**  (n=1599) | | **No Asthma**  (n=1555) | **Asthma**  (n=362) |
| --- | --- | --- | --- | --- | --- | --- |
|  | **Coefficient (95%CI)** | **p-value** | **Coefficient (95%CI)** | **p-value** | **Interaction**  **p-value** | **Interaction**  **p-value** |
| **Z-score**  **FEV_1_** | 0.04 (-0.23, 0.31) | p=0.769 | -0.02(-0.15, 0.11) | p=0.763 | p=0.519 | p=0.872 |
| **Z-score**  **FVC** | 0.02 (-0.26, 0.31) | p=0.871 | -0.01 (-0.14, 0.12) | p=0.888 | p=0.596 | p=0.851 |
| **Z-score**  **FEV_1_/FVC** | 0.00 (-0.29, 0.28) | p=0.989 | -0.03 (-0.16, 0.09) | p=0.604 | p=0.906 | p=0.679 |
| **Z-score**  **FEF_25-75_** | -0.11 (-0.39, 0.16) | p=0.427 | -0.16 (-0.29, -0.03) | **p=0.019** | p=0.819 | p=0.515 |

* Adjusted for gestation, breast feeding, siblings, passive smoke exposure, socioeconomic class, family allergy and maternal ethnicity

**Asthma defined as a positive response on questionnaire at age 6 years: Has child ever had asthma

**Supplementary Table 3:** **Association between Respiratory Infection at age 1 year and PRE-Bronchodilator lung function outcomes (absolute change) at age 10 years, stratified by food allergen sensitization at age 1 year**

|  | **Sensitized**  (n=310) | | **Not Sensitized**  (n=1312) | | **Interaction**  (n=1622) |
| --- | --- | --- | --- | --- | --- |
|  | **Coefficient (95%CI)** | **p-value** | **Coefficient (95%CI)** | **p-value** | **p-value** |
| **FEV_1 (mls)_** | -66.90 (-134.03, 0.23) | p=0.051 | -34.65 (-65.98, -3.33) | **p=0.030** | p=0.415 |
| **FVC (mls)** | -45.42 (-116.15, 25.30) | p=0.207 | -16.76 (-51.22, 17.70) | p=0.340 | p=0.488 |
| **FEV_1_/FVC (%)** | -1.33 (-2.90, 0.23) | p=0.095 | -0.92 (-1.64, -0.20) | **p=0.012** | p=0.666 |
| **FEF_25-75_ (mls/sec)** | -173.08 (-326.31, -19.85) | **p=0.027** | -99.77 (-172.26, -27.29) | **p=0.007** | p=0.465 |

* Adjusted for age, height, gestation, breast feeding, siblings, gender, passive smoke exposure, socioeconomic class, family allergy and maternal ethnicity

**Supplementary Table 4:** **Association between Respiratory Infection at age 1 year and POST-Bronchodilator lung function outcomes (absolute change) at age 10 years, stratified by food allergen sensitization at age 1 year**

|  | **Sensitized**  (n=285) | | **Not Sensitized**  (n=1179) | | **Interaction**  (n=1464) |
| --- | --- | --- | --- | --- | --- |
|  | **Coefficient (95%CI)** | **p-value** | **Coefficient (95%CI)** | **p-value** | **p-value** |
| **FEV_1 (mls)_** | -68.05 (-138.13, 2.02) | p=0.057 | -27.64 (-60.64, 5.36) | p=0.101 | p=0.300 |
| **FVC (mls)** | -59.55 (-133.24, 14.13) | p=0.113 | -9.86 (-45.21, 25.48) | p=0.584 | p=0.206 |
| **FEV_1_/FVC (%)** | -0.62 (-2.04, 0.79) | p=0.386 | -0.83 (-1.51, -0.15) | **p=0.017** | p=0.714 |
| **FEF_25-75_ (mls/sec)** | -157.58 (-312.83, -2.33) | **p=0.047** | -106.27 (-187.63, -24.91) | **p=0.011** | p=0.674 |

* Adjusted for age, height, gestation, breast feeding, siblings, gender, passive smoke exposure, socioeconomic class, family allergy and maternal ethnicity

**Supplementary Table 4B:** **Association between Respiratory Infection at age 1 year and POST-Bronchodilator lung function outcomes (z- scores) at age 10 years, in relation to history of asthma and stratified by food allergen sensitization at age 1 year**

|  | **Sensitized**  (n=285) | | **Not Sensitized**  (n=1178) | | **No Asthma**  (n=1139) | **Asthma**  (n=323) |
| --- | --- | --- | --- | --- | --- | --- |
|  | **Coefficient (95%CI)** | **p-value** | **Coefficient (95%CI)** | **p-value** | **Interaction**  **p-value** | **Interaction**  **p-value** |
| **Z-score**  **FEV_1_** | -0.25 (-0.55, 0.05) | p=0.108 | -0.12 (-0.26, 0.02) | p=0.081 | p=0.727 | p=0.628 |
| **Z-score**  **FVC** | -0.18 (-0.46, 0.10) | p=0.200 | -0.04 (-0.17, 0.08) | p=0.501 | p=0.820 | p=0.310 |
| **Z-score**  **FEV_1_/FVC** | -0.10 (-0.35, 0.15) | p=0.442 | -0.14 (-0.26, -0.02) | **p=0.025** | p=0.900 | p=0.368 |
| **Z-score**  **FEF_25-75_** | -0.27 (-0.56, 0.02) | p=0.064 | -0.20 (-0.34, -0.05) | **p=0.007** | p=0.832 | p=0.993 |

* Adjusted for gestation, breast feeding, siblings, passive smoke exposure, socioeconomic class, family allergy and maternal ethnicity

**Asthma defined as a positive response on questionnaire at age 10 years: Has child ever had asthma

**Supplementary Table 5:** **Association between Respiratory Infection at age 1 year and POST-Bronchodilator lung function outcomes (z- scores) at age 6 years, stratified by HDM allergen sensitization at age 1 year**

|  | **Sensitized**  (n=46) | | **Not Sensitized**  (n=452) | | **Interaction**  (n=498) |
| --- | --- | --- | --- | --- | --- |
|  | **Coefficient (95%CI)** | **p-value** | **Coefficient (95%CI)** | **p-value** | **p-value** |
| **Z-score**  **FEV_1_** | -0.25 (-1.13, 0.63) | p=0.568 | 0.11 (-0.13, 0.35) | p=0.358 | p=0.543 |
| **Z-score**  **FVC** | -0.24 (-1.18, 0.69) | p=0.600 | -0.11 (-0.14, 0.36) | p=0.387 | p=0.544 |
| **Z-score**  **FEV_1_/FVC** | -0.08 (-0.97, 0.80) | p=0.852 | -0.01 (-0.27, 0.25) | p=0.947 | p=0.924 |
| **Z-score**  **FEF_25-75_** | -0.18 (-1.05, 0.68) | p=0.667 | -0.07 (-0.32, 0.19) | p=0.615 | p=0.882 |

* Adjusted for gestation, breast feeding, siblings, passive smoke exposure, socioeconomic class, family allergy and maternal ethnicity

**Supplementary Table 5B:** **Association between Respiratory Infection at age 1 year and POST-Bronchodilator lung function outcomes (z- scores) at age 10 years, stratified by HDM allergen sensitization at age 1 year**

|  | **Sensitized**  (n=32) | | **Not Sensitized**  (n=366) | | **Interaction**  (n=398) |
| --- | --- | --- | --- | --- | --- |
|  | **Coefficient (95%CI)** | **p-value** | **Coefficient (95%CI)** | **p-value** | **p-value** |
| **Z-score**  **FEV_1_** | 0.08 (-1.08, 1.24) | p=0.889 | -0.30 (-0.56, -0.03) | **p=0.030** | p=0.263 |
| **Z-score**  **FVC** | 0.04 (-1.23, 1.31) | p=0.942 | -0.16 (-0.40, 0.09) | p=0.203 | p=0.788 |
| **Z-score**  **FEV_1_/FVC** | 0.14 (-0.81, 1.09) | p=0.764 | -0.20 (-0.42, 0.02) | p=0.072 | p=0.066 |
| **Z-score**  **FEF_25-75_** | -0.37 (-1.29, 0.56) | p=0.424 | -0.35 (-0.61, -0.09) | **p=0.008** | p=0.359 |

* Adjusted for gestation, breast feeding, siblings, passive smoke exposure, socioeconomic class, family allergy and maternal ethnicity
